# Evaluation of a hand-held far-ultraviolet radiation device for decontamination of Clostridium difficile and other healthcare-associated pathogens

**DOI:** 10.1186/1471-2334-12-120

**Published:** 2012-05-16

**Authors:** Michelle M Nerandzic, Jennifer L Cadnum, Kevin E Eckart, Curtis J Donskey

**Affiliations:** 1 Research Service, Louis Stokes Cleveland Veterans Affairs Medical Center, Cleveland, OH, USA; 2 Geriatric Research, Education and Clinical Center 1110 (W), Cleveland VA Medical Center, 10701 East Blvd., Cleveland, OH, 44106, USA

## Abstract

**Background:**

Environmental surfaces play an important role in transmission of healthcare-associated pathogens. There is a need for new disinfection methods that are effective against *Clostridium difficile* spores, but also safe and rapid. The Sterilray™ Disinfection Wand device is a hand-held room decontamination technology that utilizes far-ultraviolet radiation (185-230 nm) to kill pathogens.

**Methods:**

We examined the efficacy of disinfection using the Sterilray device in the laboratory, in rooms of hospitalized patients, and on surfaces outside of patient rooms (i.e. keyboards and portable medical equipment). Cultures for *C. difficile*, methicillin-resistant *Staphylococcus aureus* (MRSA), and vancomycin-resistant *Enterococcus* (VRE) were collected from commonly-touched surfaces before and after use of the Sterilray device.

**Results:**

On inoculated surfaces in the laboratory, application of the Sterilray device at a radiant dose of 100 mJ/cm^2^ for ~ 5 seconds consistently reduced recovery of *C. difficile* spores by 4.4 CFU log_10_, MRSA by 5.4 log_10_CFU and of VRE by 6.9 log_10_CFU. A >3 log_10_ reduction of MRSA and VRE was achieved in ~2 seconds at a lower radiant dose, but killing of *C. difficile* spores was significantly reduced. On keyboards and portable medical equipment that were inoculated with *C. difficile* spores, application of the Sterilray device at a radiant dose of 100 mJ/cm^2^ for ~ 5 seconds reduced contamination by 3.2 log_10_CFU. However, the presence of organic material reduced the lethal effect of the far-UV radiation. In hospital rooms that were not pre-cleaned, disinfection with the Sterilray device significantly reduced the frequency of positive *C. difficile* and MRSA cultures (*P* =0.007).

**Conclusions:**

The Sterilray™ Disinfection Wand is a novel environmental disinfection technology that rapidly kills *C. difficile* spores and other healthcare-associated pathogens on surfaces. However, the presence of organic matter reduces the efficacy of far-UV radiation, possibly explaining the more modest results observed on surfaces in hospital rooms that were not pre-cleaned.

## Background

Environmental surfaces play an important role in transmission of healthcare-associated pathogens such as *Clostridium difficile*, methicillin-resistant *Staphylococcus aureus* (MRSA), and vancomycin-resistant *Enterococcus* (VRE) [1-6]. In addition to high-touch sites inside patient rooms, contamination of portable equipment has been implicated as a source of pathogen transmission [7]. *C. difficile* is particularly challenging for infection control because it produces spores that are resistant to killing by most disinfectants [8]. Because standard cleaning methods are often suboptimal, there is a need for new environmental disinfection methods that are effective against a wide range of pathogens including *C. difficile* spores [5,6,9,10].

Several recent studies have demonstrated that an automated ultraviolet-C (UV-C) device may be effective as an adjunctive method for disinfection of healthcare-associated pathogens including *C. difficile* in patient rooms [11]. While the UV-C device has some potential advantages over other disinfection strategies, cycles that are effective for killing of *C. difficile* spores require approximately 45 minutes and patients cannot be in the room during use of the device. The mechanism of killing of microorganisms by UV-C (230–280 nm) is primarily due to inactivation of DNA through absorption of photons [12-15]. The far-UV radiation spectrum (185–230 nm) has more photon energy than UV-C, and could potentially achieve lethal doses of radiation in less time [16]. In addition to inactivation of DNA, far-UV's increased photon energy is absorbed by peptide and disulfide bonds, which breaks the bonds and causes irreparable damage [17]. However, limited data are available regarding the effectiveness of far-UV in killing healthcare-associated pathogens and it is unclear if far-UV will be effective or practical to use in healthcare settings.

The Sterilray™ Disinfection Wand (Healthy Environment Innovations, Inc., Dover, New Hampshire) is a mobile, hand-held device that utilizes far-UV radiation to kill pathogens. The far-UV administered by the Sterilray device is localized under the wand and it has been proposed that it may be used to treat surfaces in rooms occupied by patients. Here, we examined the effectiveness of the Sterilray device for killing pathogens in the laboratory and in rooms of hospitalized patients. We additionally assessed the use of the Sterilray device for reducing levels of *C. difficile* spores on computer keyboards and portable medical equipment.

## Materials and methods

The study protocol was approved by the Veterans Affairs Medical Center's research and development committee. There was no direct contact with human subjects in this study, therefore the hospital's institutional review board exempted the study protocol from review.

### Setting

The Cleveland Veterans Affairs Medical Center is a 265-bed acute care hospital. At the time of the study, active surveillance for MRSA carriage was performed and colonized or infected patients were placed in contact precautions. Patients with CDI were placed in contact precautions until they completed treatment and diarrhea resolved. No active surveillance was performed for VRE, and VRE-colonized or infected patients were not placed in contact precautions.

### *C. difficile*, MRSA, and VRE strains

One *C. difficile* strain from the American Type Culture Collection (ATCC) and 3 strains cultured from patients with CDI in Cleveland were studied. ATCC 43593 is a non-toxigenic strain of *C.difficile* from serogroup B. VA 17 is a restriction endonuclease analysis (REA) type BI strain and VA 11 and VA 9 are REA type J strains. Two clinical MRSA strains were studied, including one pulsed-field gel electrophoresis type USA300 (community-associated) strain and one USA800 strain (hospital-associated). Two VRE strains were studied, including one VanA-type isolate (C37) and one VanB-type isolate (C68).

### Preparation of *C. difficile* spores, MRSA, and VRE strains

*C. difficile* spores were prepared by growth on Duncan and Strong agar medium as previously described [18,19]. Spores were stored at 4°C in sterile distilled water until use. Prior to testing, spore preps were confirmed by phase contrast microscopy and malachite green staining to be > 99% dormant, bright-phase spores. MRSA and VRE strains were streaked for isolation and incubated for 24 hours on blood agar plates. Several colonies from overnight cultures were suspended in phosphate-buffered saline (PBS) or nutrient broth with 10 mg/ml bovine serum albumin. Colony forming units (CFU) were enumerated by serially diluting and plating suspensions on selective agar as described below in *Microbiology*.

### The Sterilray device

**Figure 1:**
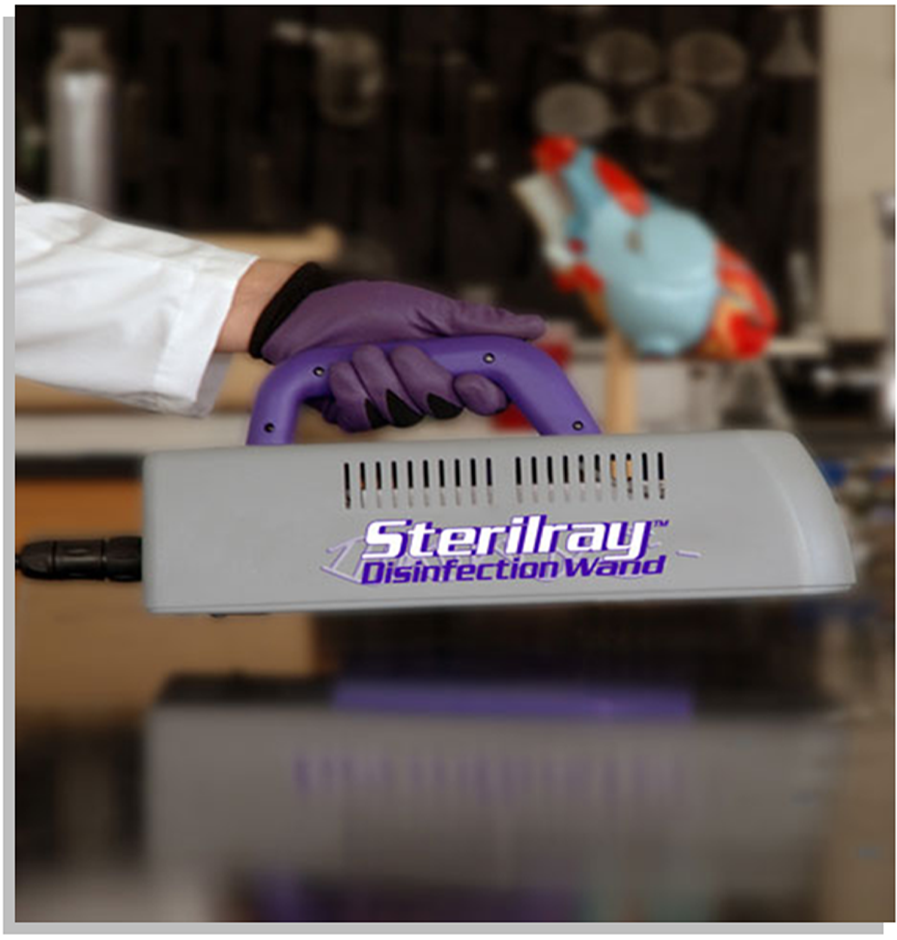
**Picture of the Sterilray**™ **Disinfection Wand far-ultraviolet radiation device.** The portable device includes a disinfecting wand weighing 4 pounds attached to a wheeled power pack. The radiant dose (mJ/cm^2^) of far-ultraviolet irradiation is dependent upon the amount of time and the distance the device is held from the surface being treated.

The Sterilray is a shielded far-UV irradiation device manufactured by Healthy Environment Innovations, Inc. It is a portable unit that contains a disinfecting wand weighing 4 pounds that is attached to a wheeled power pack (Figure 1). The far-UV irradiation emitted by the device is confined to directly below the wand. The wand is operated by a single person wearing personal protective apparel consisting of gloves, lab coat and safety glasses. The wand is held above the surface to be disinfected and activated using power buttons located on the back of the wand. There are three settings with increasing power levels, termed "smooth, texture, and deodorize". The "deodorize" setting outputs high-level photon energy with concomitant production of ozone at levels within the Food and Drug Administration (FDA) requirement that ozone output from indoor medical devices to be no higher than 0.05 parts per million [20]. Only the area directly below the wand has any measurable ozone and it deteriorates rapidly to achieve safe levels around the device (manufacturer’s unpublished data). The radiant dose (mJ/cm^2^) of far-UV is dependent upon the amount of time and the distance the device is held from the surface being treated.

### Reduction of pathogens by the Sterilray device

Initial experiments were conducted to examine the efficacy of the Sterilray device for killing of *C. difficile*, MRSA and VRE in the laboratory. Organisms were suspended in sterile phosphate buffered saline or Brain-heart infusion broth containing 10 mg/mL bovine serum albumin (simulated organic load). Nine droplets (twenty μL) of each organism (10^6^ to 10^8^ CFU) were inoculated onto the cover of a sterile, plastic Petri dish. To assess the lethal effects of increasing doses of far-UV and to determine the effects of concomitant production of ozone at the high power setting, the droplets were either subjected to low power ("smooth" setting) or high power ("deodorize" setting with ozone) irradiation at a radiant dose of 10, 20, 30, 50 or 100 mJ/cm^2^ with the Sterilray device or left untreated (i.e. positive controls). Radiant dose was measured by placing a radiometer (International Light, Inc., Newburyport, MA, USA) next to the samples during treatment with Sterilray. After the organisms were treated, the droplets were collected by tipping the Petri dish cover and collecting the drops into a tube. The organisms were enumerated by serially diluting and plating suspensions. Experiments were repeated three times.

Additional experiments were performed to test the efficacy of the Sterilray device for killing spores in the presence of naturally-occurring organic material. Stool samples, containing vegetative and spore forms of *C.difficile*, were obtained from two patients with active CDI and 10 microliter aliquots (10^3^-10^4^ CFU) were spread in thin layers onto 2 cm^2^ areas of glass slides. Previously it has been demonstrated that vegetative forms of *C.difficile* die rapidly upon desiccation in room air [21]. Therefore, to confirm that only spores remained, the slides were allowed to desiccate in room air for an hour to eliminate vegetative *C.difficile,* then subjected to high power irradiation at a radiant dose of 100 mJ/cm^2^ with the Sterilray device or left untreated. The slides were sampled with sterile swabs (BD BBL™ CultureSwab™, Becton Dickinson, Cockeysville, MD) pre-moistened with saline, transferred into an anaerobic chamber, spread onto pre-reduced *C. difficile* selective media and incubated as described previously. Colony forming units were enumerated using Fotodyne's TotalLab Quant Analysis software (Fotodyne Inc., Hartland, Wisconsin) and treated versus untreated samples were compared.

### Reduction of *C. difficile* on keyboards and portable medical equipment

Experiments were performed to assess the use of the Sterilray device for reduction of *C. difficile* spores artificially applied to keyboards and portable medical equipment (i.e. stethoscopes, pulse oximeters, blood pressure cuffs). Ten microliters of ATCC 43593 *C. difficile* spores (10^4^ to 10^5^ CFU) were inoculated onto a 1 cm^2^ area of each surface. After air drying, each object was subjected to a high power radiant dose of 100 (mJ/cm^2^) for ~5 seconds or left untreated (positive control). The inoculated surfaces were then sampled with sterile swabs (BD BBL™ CultureSwab™, Becton Dickinson, Cockeysville, MD) pre-moistened with saline, transferred into an anaerobic chamber, spread onto pre-reduced *C. difficile* selective media and incubated as described previously. Colony forming units were enumerated using Fotodyne's TotalLab Quant Analysis software (Fotodyne Inc., Hartland, Wisconsin) and treated versus untreated samples were compared. Experiments were performed in triplicate.

### Disinfection of surfaces in hospital rooms

The efficacy of the Sterilray device was assessed in rooms of discharged patients that had not yet been cleaned by housekeeping. Organisms were not inoculated onto surfaces for these experiments. Sterile swabs (BD BBL™ CultureSwab™, Becton Dickinson, Cockeysville, MD) pre-moistened with saline were used to collect cultures from the call light, bedside table, telephone, and bed rail before and after use of the Sterilray device on high power at a radiant dose of 100 mJ/cm^2^ for ~ 5 seconds. MRSA cultures were collected from MRSA isolation rooms, whereas VRE and *C. difficile* cultures were collected from rooms that housed patients with CDI and from non-CDI rooms. The device was held 10 cm above each surface during disinfection. For the bed rail and table, 5 x 20 cm areas were cultured before Sterilray disinfection and adjacent areas of the same surface area were cultured after disinfection; for the call button and telephone, half of the entire surface area (~5 x 10 cm for the call button and ~5 x 20 cm for the telephone) was cultured before Sterilray disinfection and the other half was cultured after disinfection.

### Microbiology

For VRE, MRSA, and *C. difficile* cultures, selective media included Enterococcosel agar (Becton Dickinson, Cockeysville, MD) containing 20 μg/mL of vancomycin, CHROMagar (Becton Dickinson) containing 6 μg/mL of cefoxitin, and cycloserine-cefoxitin-brucella agar containing 0.1% taurocholic acid and lysozyme 5 mg/mL (CDBA), respectively [16]. Plates were incubated at 37°C for 48 hours. VRE and MRSA colonies with unique morphology were subjected to identification and susceptibility testing in accordance with Clinical Laboratories Standards Institute guidelines [17]. *C. difficile* was confirmed on the basis of typical odor and appearance of colonies and by a positive reaction using *C. difficile* latex agglutination (Microgen Bioproducts, Camberly, UK).

### Statistical analysis

Data were analyzed using STATA 9.0 (StataCorp, College Station, TX). Continuous data were analyzed using paired *t* tests and categorical data were assessed using Fisher’s exact test.

## Results

### Reduction of pathogens by the Sterilray device

**Figure 2:**
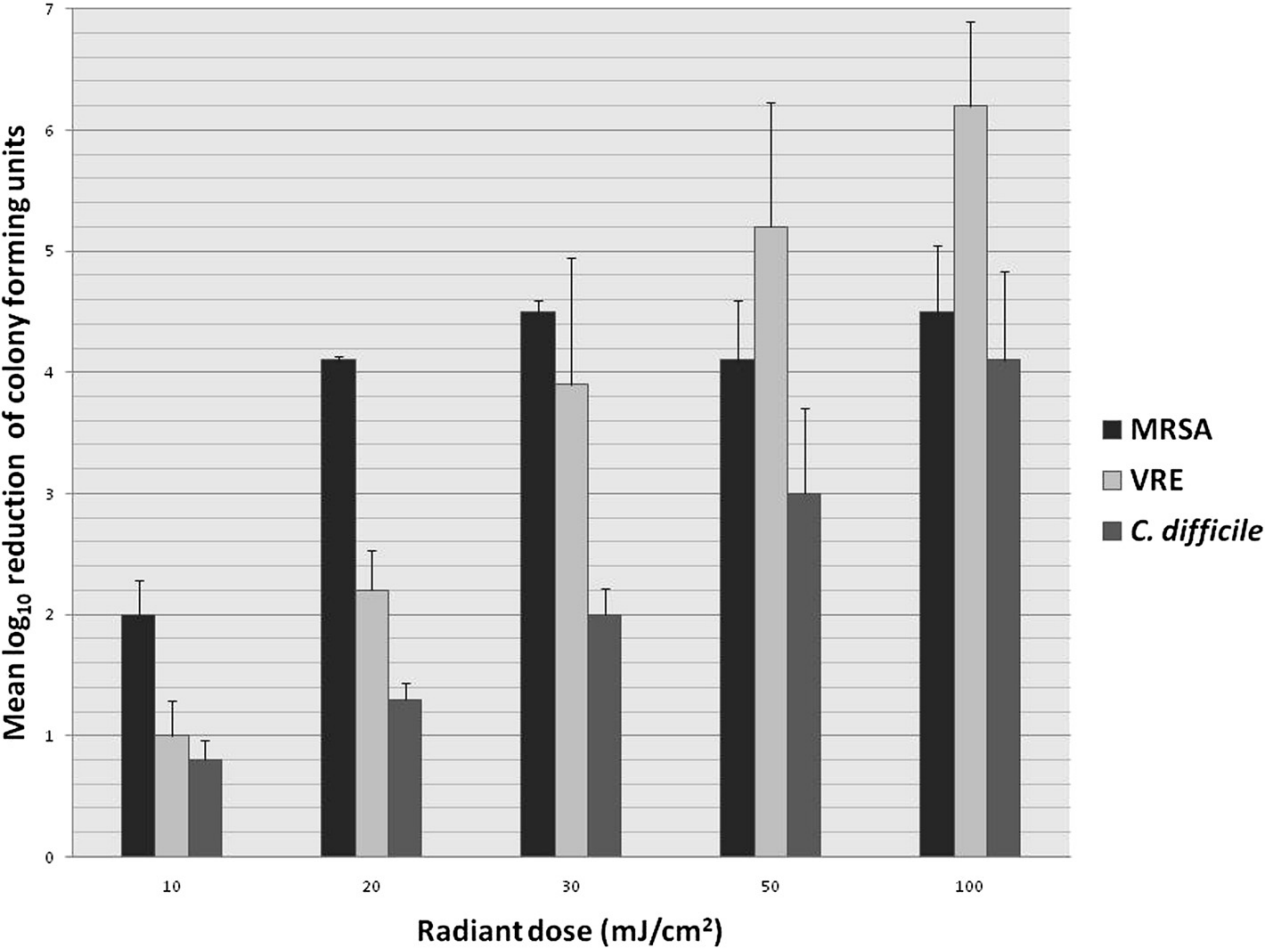
**Mean reduction (log**_**10**_**colony-forming units (CFU)/cm**^**2**^**in recovery of multiple strains of*Clostridium difficile*spores, methicillin-resistant*Staphylococcus aureus*(MRSA), and vancomycin-resistant*Enterococcus*(VRE) suspended in phosphate-buffered saline.** The Sterilray far-ultraviolet irradiation device was operated at radiant doses ranging from 10 to 100 mJ/cm^2^ for ~5 seconds.

Figure 2 shows the mean log_10_ reduction of recovery of each organism after increasing doses of radiation with the Sterilray device. On inoculated surfaces, application of the Sterilray device at a radiant dose of 100 mJ/cm^2^ for ~ 5 seconds consistently reduced *C. difficile* spores by 4.4 log_10_CFU, MRSA by 5.4 log_10_CFU and of VRE by 6.9 log_10_CFU. MRSA and VRE were reduced by >3 logs when the radiant dose was lowered to 30 mJ/cm^2^ for ~ 2 seconds, however killing of *C. difficile* was reduced. The ozone generated at the high power setting ("deodorize" setting) did not significantly increase the lethal effects of the Sterilray device (data not shown).

When pathogens were suspended in brain-heart infusion broth with 10 mg/ml bovine serum albumin the lethal effects of the Sterilray device were completely eliminated (i.e., no killing of pathogens was observed in the presence of the heavy simulated organic load). C*. difficile* spores in a fecal matrix were reduced by ~1 log_10_CFU after treatment with the Sterilray device at a radiant dose of 100 mJ/cm^2^ for ~5 seconds.

### Reduction of *C. difficile* on keyboards and portable medical equipment

On keyboards and portable medical equipment that were inoculated with *C.difficile* spores suspended in sterile water, application of the Sterilray device at a radiant dose of 100 mJ/cm^2^ for ~ 5 seconds consistently reduced contamination by 3.2 log_10_CFU (*P* < 0.001).

### Disinfection in hospital rooms

Table 1 provides a summary of the results of disinfection of pathogens in hospital rooms using the Sterilray device. For MRSA (n =27 rooms and 106 culture sites) and *C. difficile* (n =67 rooms and 265 culture sites), there was a significant reduction in the percentage of contaminated sites. For VRE (n =67 rooms and 267 sites cultured), the frequency of contamination prior to disinfection was lower than for the other pathogens; Sterilray disinfection reduced the percentage of sites contaminated with VRE, but the reduction was not statistically significant. The mean number of colonies of each pathogen recovered from contaminated surfaces was reduced after disinfection with the Sterilray device, but the differences were not statistically significant.

**Table 1:** **Recovery of*Clostridium difficile*, vancomycin-resistant*Enterococcus*, and methicillin-resistant*Staphylococcus aureus*(MRSA) from surfaces in hospital rooms before and after use of the Sterilray device**

	**Sites positive(%)**			**Mean colony forming units recovered (range)**		
	***C.difficile***	**VRE**	**MRSA**	***C. difficile***	**VRE**	**MRSA**
**Before Sterilray**	28 (11)	12 (5)	49 (46)	0.23(0–29)	0.47(0–43)	28.2(0–1000)
**After Sterilray**	11 (4)	5 (2)	29 (27)	0.06(0–2)	0.28(0–56)	7.84(0–200)
***P***	0.007	0.14	0.007	0.71	0.57	0.15

## Discussion

The Sterilray far-UV device consistently and rapidly killed *C. difficile* spores, MRSA, and VRE in the absence of organic material (i.e. artificial inoculation in the laboratory setting and on keyboards and portable equipment). *C.difficile* spores were reduced by 4.4 log_10_CFU in ~5 seconds, while MRSA and VRE showed similar reductions in ~2 seconds. However, the lethal effects of the device were reduced when spores were in the presence of stool and completely eliminated in the presence of a heavy organic load (i.e., nutrient broth and 10 mg/ml bovine serum albumin). This phenomenon may explain the fact that the Sterilray device had relatively modest effects in reducing levels of pathogens on surfaces in hospital rooms that had not previously been cleaned. These results suggest that the Sterilray device could be used as an adjunct to routine cleaning measures in healthcare facilities, but that it will be most effective in settings where organic load is minimized.

The Sterilray device has some potential advantages over other disinfection technologies. First, it provides very rapid killing of pathogens. The Sterilray device kills *C. difficile* spores on surfaces in seconds, whereas UV-C room disinfection devices may require 45 minutes and hydrogen peroxide vapor or mist systems may require hours for setup and disinfection of a hospital room [11,22,23]. Second, according to the manufacturer, the Sterilray device can safely be used when patients are in the room. Third, there were no apparent adverse effects on surfaces when the Sterilray device was used repeatedly ( >10 applications), whereas sporicidal disinfectants such as sodium hypochlorite may be corrosive to materials. Fourth, the Sterilray is portable and can be transported easily from room to room or to areas where portable equipment and keyboards are located. Moreover, the device could potentially fill a niche for reducing pathogens on objects that are not easily or frequently disinfected (i.e. plastic/vinyl curtains, stethoscopes, shoes, patient belongings, etc.). Finally, the Sterilray device is relatively easy to use and does not require a dedicated staff to operate. Therefore, after the initial purchase of the device (~ $30,000), the cost of operating and maintaining the Sterilray device is minimal.

The Sterilray device does have some potential limitations. Far-UV irradiation does not penetrate porous fabrics. Therefore, it will not be effective for disinfecting items such as bedding, cloth curtains, or upholstery. Additionally, the presence of organic material on surfaces will reduce the effectiveness of far-UV radiation. Further studies are needed to determine if pre-cleaning surfaces with a detergent product will improve the effectiveness of the Sterilray device. Our assessment was limited to research workers rather than housekeeping staff. Additional studies are needed to evaluate whether the far-UV device can easily be incorporated into house-keeping practices. The device is not automated and correct usage by the operator with regard to application time and distance from the surfaces is required. Development of a far-UV box for disinfection of portable equipment and devices is in progress and would eliminate the potential for operator error by providing standardized exposure to far-UV.

Our study has some limitations. The use of swabs and direct plating to quantify the concentrations of bacteria on surfaces is imprecise at higher concentrations. In addition, recovery and release of bacteria from swabs is less than 100% and therefore we may not have detected lower levels of bacteria on surfaces. However, methods were standardized for processing all samples so any limitations in the methodology would be equally shared by baseline and experimental groups. Environmental surfaces collected with the use of swabs were not blinded to lab personnel, therefore there is the additional possibility of sampling bias in this study. However, for simplicity purposes, each surface was split into half, and pre and post-disinfection surfaces were not varied in this study in order to avoid errors when culturing and processing the samples.

## Conclusions

The Sterilray far-UV Disinfection Wand is a novel environmental disinfection technology that rapidly kills *C. difficile* spores and other healthcare-associated pathogens on surfaces. However, the presence of organic matter decreases the efficacy of far-UV radiation, and may limit the utility of this technology in healthcare settings. Further studies are needed to determine if the device may be useful as an adjunct to standard environmental cleaning measures in healthcare facilities.

## Competing interests

The authors declare that they have no competing interests related to this article.

## Authors’ contributions

MMN contributed to the study design, supervised the data collection and culture processing, participated in drafting the manuscript, and participated in editing the manuscript. JLC participated in data collection. KEE participated in data collection. CJD contributed to study design, data analysis, and drafting and editing of the manuscript. All authors read and approved the final manuscript.

## Pre-publication history

The pre-publication history for this paper can be accessed here:

http://www.biomedcentral.com/1471-2334/12/120/prepub
